# A 3-dimensional airway model for tracheobronchial surgery

**DOI:** 10.1016/j.xjtc.2022.01.024

**Published:** 2022-02-21

**Authors:** Kohei Hashimoto, Kenshiro Omura, Junji Ichinose, Yosuke Matsuura, Masayuki Nakao, Mingyon Mun

**Affiliations:** Department of Thoracic Surgical Oncology, Cancer Institute Hospital, Japanese Foundation for Cancer Research, Tokyo, Japan

**Figure:**
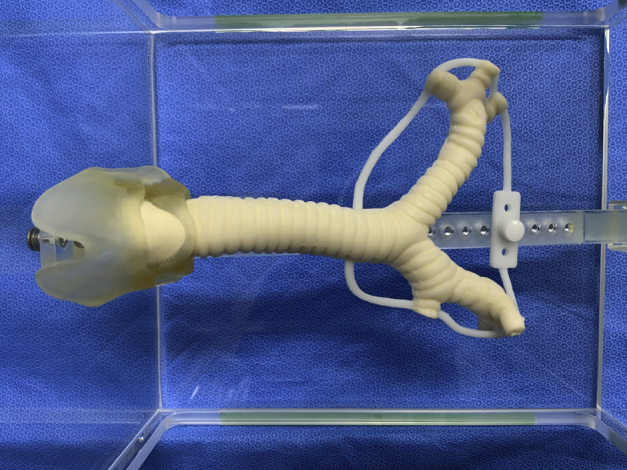
Interactive 3D airway model designed based on human chest computed tomography.

**Central Message**

A 3D airway model with precise human anatomy and texture was successfully created. Tracheobronchial reconstruction procedures were reproduced in the model, showing its promise in surgical simulation.

Airway reconstruction procedures, such as sleeve bronchial or tracheal resection, are established surgical techniques that are occasionally required to achieve complete resection of bronchopulmonary malignancies that invade the central airway while preserving the pulmonary reserve. The number of patients with bronchopulmonary malignancies requiring this procedure appears to have decreased at least in our country^1^ and perhaps in other countries too. However, it is still critical for all thoracic surgeons to acquire this procedure to perform an oncologically sound operation while reducing postoperative complications. These skills are also important for surgical benign airway diseases. The interactive 3-dimensional (3D) airway model was designed to assist with training for the tracheobronchial surgical technique.

## Methods

Data of noncontrast chest computed tomography (CT) (slice thickness, 1 mm) performed on a healthy male volunteer (one of the authors) during a health check-up before the study period was utilized. The Digital Imaging and Communications in Medicine data were converted to 3D data (OsiriX MD version 12.0; Pixmeo). The airway structure was determined, and the cartilage was distinguished from other connective tissues (Geomagic Freeform; 3D Systems). The data were then converted to Standard Triangle Language format for 3D printing. The hard plastic models of the cartilage and other connective tissues were 3D printed using a stereolithography method (SCS-8100; Sony Manufacturing Systems). These plastic models served as frameworks for the creation of silicone molds. Two urethan materials mimicking cartilage (Hapla Pudding Gel-PL00; Polysis) and the remaining connective tissue (Adapt, RU-843A-N80; Nisshin Resin) were poured into the molds while the 2 parts were combined using a vacuum casting method (CrossEffect). An airway model consisting of multiple soft materials was created in this manner. A holder was also created to simulate surgical exposure of the airway designed based on the CT measurements of the healthy volunteer. Right upper sleeve lobectomy and tracheal sleeve resection were then performed on the airway model. This study was approved by the Institutional Ethical Review Board (No. 2020-GA-1334) on May 28, 2021, and consent was waived because of its retrospective nature.

## Results

**Figure 1:**
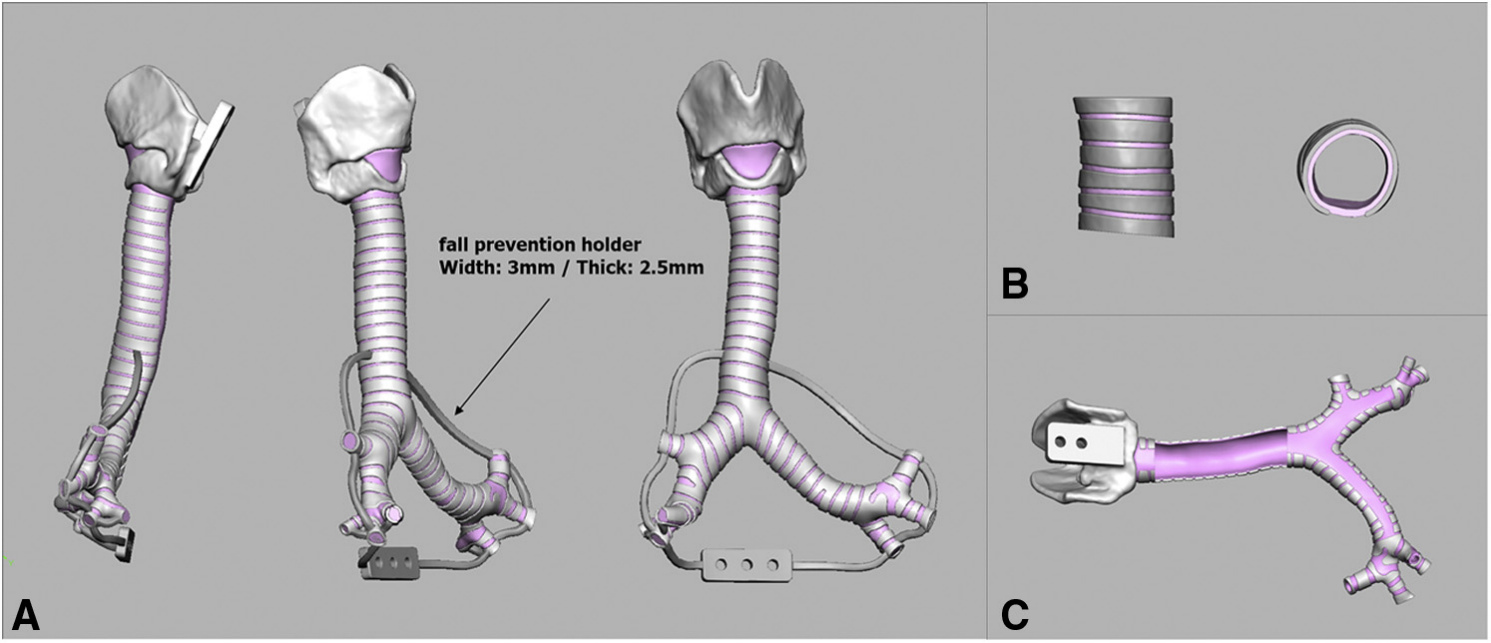
Design of a 3-dimensional airway model using data obtained during chest computed tomography. A, An overall view. A fall prevention holder was attached to avoid part of the model falling during procedure. B, A magnified image and an axial view. C, A back view showing the area of membranous portion. Note that white and pink areas were recognized separately, and 2 different urethans were utilized to represent airway anatomy.

**Figure 2:**
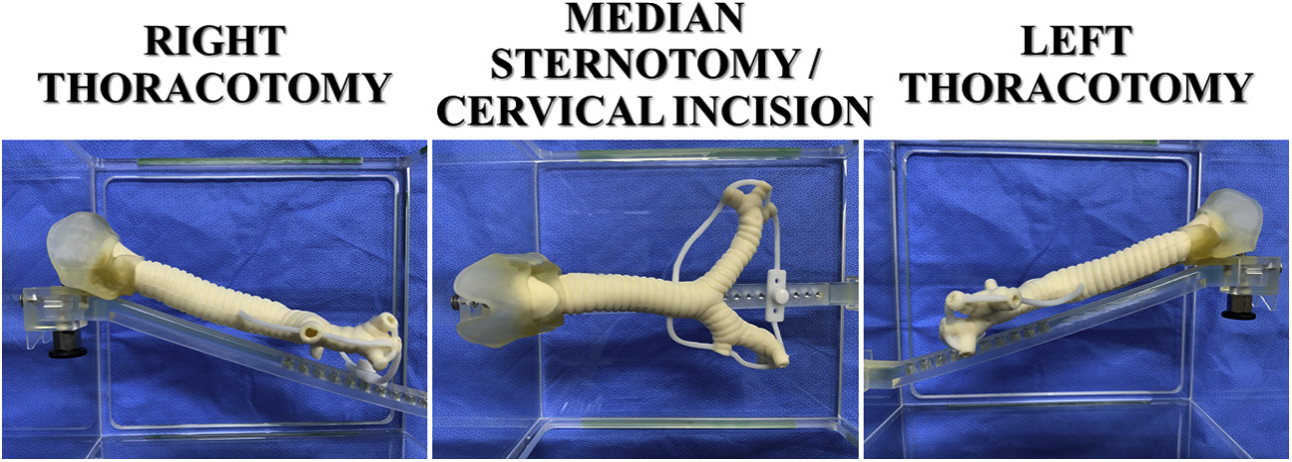
A 3-dimensional airway model consisting of multiple urethan materials mimicking cartilage and the remaining connective tissue. A special holder allows the mimicking of surgical exposures of the airway.

A tissue-like 3D airway model of the area extending from the larynx to the segmental bronchus was successfully created (Figure 1). The surgical exposures of the airway through a right and left thoracotomy and median sternotomy or cervical incision were reproduced by mounting the model on the holder (Figure 2).

Four board-certified thoracic surgeons rated the model based on how closely it simulated the exposure and tissue texture of the airway to that observed during an actual surgery (5-point Likert scale where 1 = poor, 3 = moderate, and 5 = excellent). The averages scores were generally acceptable: surgical exposure, 3.8 (range, 3-5); rigidity, 3.0 (range, 2-4); elasticity, 4.0 (range, 3-5); resistance to needles, 3.0 (range, 2-5); and resistance to tying 4.0 (range, 2-5).

A board-certified thoracic surgeon was able to reproduce the surgical procedures in the 3D airway model (Figure 2). The Video 1 demonstrates the appearance and texture of the airway model and compares the surgical simulation using the 3D airway model with actual tracheobronchial reconstruction procedures performed by the same thoracic surgeon. These procedures were performed consistently on the model for four consecutive sessions.

## Discussion

Surgical techniques were traditionally taught in operation theaters; however, laboratory training programs for surgical trainees have recently been integrated into training programs at institutional and societal levels to improve training efficiency along with patient safety.^2^ Laboratory training for surgical procedures that are high-risk, complex, and relatively rare may be especially important.

Existing materials for laboratory training include extirpated animal organs, living animals, cadavers, or artificial materials. However, these models are imperfect in terms of cost and anatomical reality. The advent of 3D printing technology has allowed the creation of a precise organ model.^3^ Initial 3D-printed models have been proposed for surgical planning by appreciating 3D human anatomy. Few interactive models, such as those for cardiac surgery^4^ and brain surgery^5^ have been reported in the literature to date. In this study, we created the first interactive 3D airway model to our knowledge.

This study has shown the proof of concept of an operative 3D airway model based on CT data for tracheobronchial surgical simulation. We will use this model to develop educational programs that will allow thoracic surgery trainees to gain experience with tracheobronchial procedures that are decreasing while remaining important techniques. We are also developing disease models for patient-specific simulation that would benefit even board-certified surgeons.

## Conclusions

A 3D airway model mimicking the anatomy and texture of the human airway was successfully created using CT data. Two typical tracheobronchial reconstruction procedures were reproduced in the airway model as a proof of concept of the operative 3D airway model for tracheobronchial surgical simulation.

## Supplementary Data

Video 1 Demonstration of the handling of an airway model and comparison between a right upper sleeve lobectomy and tracheal sleeve resection on the airway model and the actual surgeries performed by the same thoracic surgeon. Part of this video (the case of tracheal sleeve resection) is previously published. The video was re-edited by the author of the present study and permission was granted by the publisher (Springer Nature, License No.: 5177080294607). Video available at: https://www.jtcvs.org/article/S2666-2507(22)00106-7/fulltext.
