# Supplementary figures and images for: A 3-dimensional airway model for tracheobronchial surgery
Source: JTCVS Tech. 2022 Feb 21;13:247–9. doi: 10.1016/j.xjtc.2022.01.024 (PMC9196135; doi:10.1016/j.xjtc.2022.01.024)

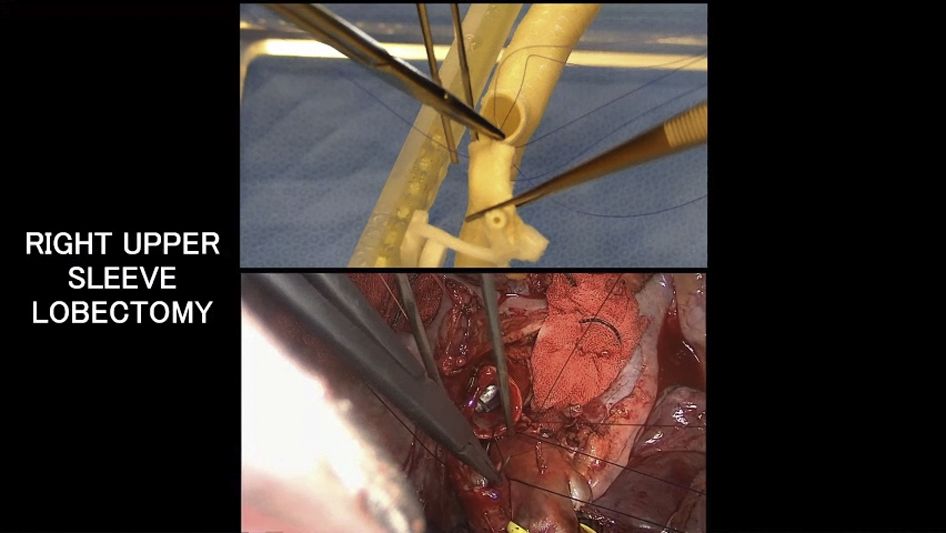

Supplement: Video 1 — Demonstration of the handling of an airway model and comparison between a right upper sleeve lobectomy and tracheal sleeve resection on the airway model and the actual surgeries performed by the same thoracic surgeon. Part of this video (the case of tracheal sleeve resection) is previously published. The video was re-edited by the author of the present study and permission was granted by the publisher (Springer Nature, License No.: 5177080294607). Video available at: https://www.jtcvs.org/article/S2666-2507(22)00106-7/fulltext. [file fx2.jpg]
